# Mouse CD146+ muscle interstitial progenitor cells differ from satellite cells and present myogenic potential

**DOI:** 10.1186/s13287-020-01827-z

**Published:** 2020-08-06

**Authors:** Bartosz Mierzejewski, Iwona Grabowska, Daniel Jackowski, Aliksandra Irhashava, Zuzanna Michalska, Władysława Stremińska, Katarzyna Jańczyk-Ilach, Maria Anna Ciemerych, Edyta Brzoska

**Affiliations:** Department of Cytology, Faculty of Biology, University of Warsaw, Miecznikowa 1 St, 02-096 Warszawa, Poland

**Keywords:** Mouse, Skeletal muscle regeneration, Differentiation, Bone marrow-derived mesenchymal stem/stromal cells, Interstitial cells, Satellite cells, CD146, Nestin

## Abstract

**Background:**

The skeletal muscle regeneration relays on the satellite cells which are stem cells located between basal lamina and plasmalemma of muscle fiber. In the injured muscles, the satellite cells become activated, start to proliferate, and then differentiate into myoblasts, which fuse to form myotubes and finally myofibers. The satellite cells play the crucial role in the regeneration; however, other cells present in the muscle could also support this process. In the present study, we focused on one population of such cells, i.e., muscle interstitial progenitor cells.

**Methods:**

We used the CD146 marker to identify the population of mouse muscle interstitial cells. We analyzed the expression of selected markers, as well as clonogenic, myogenic, adipogenic, and chondrogenic potential in vitro. Simultaneously, we analyzed satellite cell-derived myoblasts and bone marrow-derived mesenchymal stem/stromal cells that allowed us to pinpoint the differences between these cell populations. Moreover, we isolated CD146+ cells and performed heterotopic transplantations to follow their in vivo differentiation.

**Results:**

Mouse muscle CD146+ interstitial progenitor cells expressed nestin and NG2 but not PAX7. These cells presented clonogenic and myogenic potential both in vitro and in vivo. CD146+ cells fused also with myoblasts in co-cultures in vitro. However, they were not able to differentiate to chondro- or adipocytes in vitro. Moreover, CD146+ cells followed myogenic differentiation in vivo after heterotopic transplantation.

**Conclusion:**

Mouse CD146+ cells represent the population of mouse muscle interstitial progenitors that differ from satellite cell-derived myoblasts and have clonogenic and myogenic properties.

## Background

The skeletal muscle regeneration covers two phases—degeneration, accompanied with necrosis and inflammation, and reconstruction. The myofiber reconstruction relies on muscle stem cells—satellite cells (SCs), which once activated proliferate, differentiate into myoblasts that fuse with each other, and form myotubes maturing into myofibers. Along these processes, vasculature and innervation of muscles are also restored. SCs have been studied for almost 60 years since they were discovered by Mauro [1]. It is widely accepted that they are necessary for skeletal muscle reconstruction [2]. Their indispensability was documented using *Pax7* null mice which were characterized by the SC deficiency and inability to regenerate injured muscle [3–5]. Also, postnatal ablation of SCs led to ineffective regeneration [6, 7]. In intact muscles, SCs are defined on the basis of their very characteristic localization, i.e., between the basal lamina and muscle fiber plasmalemma. The most important factors that are engaged in the activation and differentiation of SCs are paired/homeodomain transcription factors PAX3 and PAX7 and basic helix-loop-helix myogenic regulatory factors (MRFs) such as MYF5, MRF4, MYOD, and myogenin [8, 9]. SCs also express few characteristic surface proteins, such as m-cadherin, α7-integrin, CD34, vascular cell adhesion protein (VCAM), neural cell adhesion molecule (NCAM), syndecan3/4, CD34, and C-X-C chemokine receptor type 4 (CXCR4) [2, 10, 11].

Except for SCs, other cell types, such as fibroblasts, endothelial cells, or resident and infiltrating inflammatory cells, reside in the skeletal muscle interstitium, i.e., between myofibers and outside basal lamina, and impact the myofiber reconstruction and restoration of skeletal muscle tissue homeostasis [12]. Moreover, different populations of interstitial stem/progenitor cells were described in mouse and human skeletal muscles [12]. Some authors use the term “muscle mesenchymal stromal/stem/progenitor cells” to describe this heterogeneous population of interstitial cells. However, it should be noticed that except differences in marker expression, these cells have diverse clonogenic and differentiation potential and, as a result, the role in skeletal muscle homeostasis [12]. Among such cells are fibro-adipogenic progenitors (FAPs), characterized on the basis of platelet-derived growth factor receptor α (PDGFRα), β (PDGFRβ), CD34, stem cell antigen-1 (Sca1) expression, and presenting the ability to differentiate into fibroblasts and adipocytes [12, 13]. Importantly, FAPs secrete factors that induce differentiation of myoblasts and lack of these cells impairs skeletal muscle regeneration [14, 15]. Moreover, the interstitium is the source of other cells presenting myogenic potential, such as PW1+ interstitial cells (PICs), TWIST2+ cells, or pericytes [12]. PICs were characterized on the basis of PW1, Sca1, and CD34 presence. These cells were shown to be able to generate smooth muscles, skeletal muscles, and adipocytes [16]. The myogenic potential of PICs was shown in vitro and also in vivo, after their injection into the damaged muscle [16]. Another population of interstitial myogenic progenitors, described in mouse muscles, consists of TWIST2+ cells [17]. These cells participate in myofibers formation during skeletal muscle regeneration and effectively fuse with each other in vitro, in the absence of myoblasts [17]. Next, peripherally located to microvessel endothelium pericytes and mesoangioblasts were investigated. These cells express similar markers such as neural-glial antigen (NG2), PDGFRβ, tissue non-specific alkaline phosphatase (ALP), CD146, smooth muscle α-actin (αSMA), desmin, and nestin [18–22]. Pericyte characteristics depend greatly on their source [23]. For example, these ones residing in the skeletal muscle could be divided into two subpopulations, i.e., type 1 (nestin−/NG2+) and type 2 (nestin+/NG2+). Only type 2 pericytes were shown to be able to follow the myogenic program [24–26]. Thus, pericytes exposed to differentiation promoting medium-formed myotubes in vitro and after transplantation into damaged muscles occupied SCs niche and participated in new myofiber reconstruction [18, 19, 22, 27]. Importantly, pericytes secrete factors modulating SC quiescence and myofiber growth [21]. Moreover, Sacchetti and coworkers described the population of human CD146+ clonogenic myogenic progenitors, localized as adventitial reticular cells in the microvascular compartment in human muscles, i.e., with characteristic similar to pericytes [28]. These cells followed the myogenic program in vitro, spontaneously forming myotubes and expressing myogenic factors (PAX7 and MYF5) and myosin heavy chains, and were able to undergo myogenic differentiation after their heterotopic or orthotopic transplantation [28].

In the present study, we used the CD146 marker to isolate the population of mouse muscle interstitial progenitor cells (MIPCs). Moreover, we compared MIPCs, SC-derived myoblasts, and bone marrow-derived mesenchymal stem/stromal cells (BMSCs) in terms of their localization, clonogenic properties, expression of selected markers, and the ability to differentiate into myogenic, chondrogenic, and adipogenic line. Furthermore, we analyzed the differentiation of MIPCs in vivo after their heterotopic transplantation.

## Methods

The animal studies were approved by the Local Ethics Committee No. 1 in Warsaw, Poland (permit number 668/2018).

### Satellite cell isolation and culture

Satellite cells (SCs) were isolated from *Gastrocnemius*, *EDL*, and *Soleus* muscles of 2–3-month-old C57/BL6 male mice, according to Rosenblatt and coworkers [29]. Briefly, mice were sacrificed by cervical dislocation, muscles were isolated, cut into smaller pieces, and incubated in 0,2% collagenase type I (Sigma-Aldrich) in Dulbecco’s modified Eagle’s medium (DMEM; ThermoFisher Scientific) at 37 °C for 2 h. Single fibers were collected using pipette tips, purified twice in DMEM containing glucose 1 g/l, and supplemented with 10% horse serum (HS, ThermoFisher Scientific), 1% penicillin/streptomycin (ThermoFisher Scientific), and 0.5% chicken embryo extract (CEE, ThermoFisher Scientific). Then, single fibers were transferred and suspension of muscle fibers was passed through a syringe with 21G needle and filtered through 40 μm strainer. Obtained SCs were plated directly on culture dishes coated with Matrigel Growth Factor Reduced (GFR) Basement Membrane Matrix (Corning) or culture dishes containing cover slides coated with Matrigel GFR Basement Membrane Matrix. Cells were expanded in DMEM with glucose 1 g/l and supplemented with 10% HS, 20% FBS, 1% penicillin/streptomycin, and 0.5% CEE and cultured under standard conditions: 37 °C, 5% CO_2_. The medium was replaced every 2 days. Under such conditions, SCs started myogenic differentiation and formed SC-derived myoblasts.

### Muscle interstitial progenitor cell (MIPC) isolation, sorting, and culture

Muscle interstitial progenitor cells (MIPCs) were obtained from *Gastrocnemius*, *EDL*, and *Soleus* muscles of 2–3-month-old C57/BL6 male mice. Briefly, mice were sacrificed by cervical dislocation, and muscles were isolated, cut into smaller pieces, and incubated in 0.2% collagenase type I in DMEM at 37 °C for 2 h. Digested muscles were passed through a serological pipette and centrifuged. The supernatant was removed, dispase in DMEM solution (2 U/ml) was added, and suspension was incubated at 37 °C for 30 min. After digestion, the muscles were passed through 25 ml, 10 ml, 5 ml, and 2 ml serological pipettes to obtain homogenous suspension which was centrifuged, the supernatant was removed, the pellet was washed in phosphate buffer saline (PBS), and centrifuged again. Next, the supernatant was removed and cells expressing CD146 (CD146+ MIPCs) were selected using magnetic columns (MACS; Miltenyi Biotec) and antibody against CD146 conjugated with ferromagnetic particles, according to manufacturer’s protocol (Miltenyi Biotec). Finally, CD146+ cells were suspended in DMEM containing glucose 4.5 g/l and supplemented with 15% FBS and 1% penicillin/streptomycin and plated in culture dishes or culture dishes containing cover slides coated with 3% gelatin (Sigma-Aldrich) solution in water. Cells were expanded under standard conditions: 37 °C, 5% CO_2_. The medium was replaced every 2 days.

### Bone marrow stromal/stem cell isolation, sorting, and culture

Bone marrow stromal/stem cells (BMSCs) were obtained from femurs and tibialis bones of 2–3-month-old C57/BL6 male mice. Briefly, mice were sacrificed by cervical dislocation, and the bones were isolated, cleared from surrounding tissues, and placed into PBS. The bone marrow was rinsed from the bones with PBS and centrifuged twice. Then, the cell pellet was suspended in growth DMEM containing glucose 4.5 g/l, supplemented with 20% FBS and 1% gentamycin (ThermoFisher Scientific), and cells were plated in culture dishes. A part of isolated cells was cultured for 7 days, and then, cells expressing CD146 (CD146+ BMSCs) were selected using magnetic columns (MACS; Miltenyi Biotec) and antibody against CD146 conjugated with ferromagnetic particles, according to manufacturer’s protocol (Miltenyi Biotec). CD146 + BMSCs were plated onto cover slides covered with Matrigel GFR Basement Membrane Matrix (Sigma-Aldrich). Cells were expanded in DMEM with glucose 4.5 g/l, supplemented with 20% FBS and 1% gentamycin under standard conditions: 37 °C, 5% CO_2_. The medium was replaced every 2 days.

### Fibroblast isolation

Mouse primary fibroblasts were obtained from ears of 2–3-month-old C57/BL6 male mice. Briefly, mice were sacrificed by cervical dislocation and their ears were shaved, dissected, cut into smaller pieces, and placed in the culture dish covered with 3% gelatin (Sigma-Aldrich) solution in water DMEM with glucose 4.5 g/l, supplemented with 15% FBS and 1% penicillin/streptomycin. After 10 days of culture, tissue fragments were removed and obtained cells were further expanded under standard conditions: 37 °C, 5% CO_2_. The medium was replaced every 2 days.

### Colony-forming unit assay

Colony-forming unit (CFU) assay was performed for three examined populations: SC-derived myoblasts, CD146+ MIPCs, and BMSCs. Cells were seeded in concentration 1.6 cell/cm^2^ in DMEM containing glucose 4.5 g/l, supplemented with 20% FBS and 1% gentamycin (ThermoFisher Scientific). After 14 days of in vitro culture, they were fixed with cold methanol and stained with Giemsa (Merck), according to manufacturer’s instruction. Then, the number of colonies was counted. Three independent experiments were performed for each of the examined cell populations.

### Adipogenic, chondrogenic, and myogenic differentiation assays

The adipogenic, chondrogenic, and myogenic properties of three examined cell populations were analyzed. The spontaneous/naïve differentiation potential of CD146+ MIPCs, SC-derived myoblasts, and BMSCs was examined. After 3 days of culture of SC-derived myoblasts, 5 days of culture of CD146+ MIPCs or 7–10 days of culture of BMSCs, the culture medium was changed to the one promoting myogenic differentiation, i.e., DMEM 1 g/l supplemented with 10% HS, 20% FBS, 1% penicillin/streptomycin, and 0.5% CEE. The ability of CD146+ MIPCs to fuse in the presence of exogenous myoblasts was analyzed in co-cultures of CD146+ MIPCs and SC-derived myoblasts. Briefly, CD146+ MIPCs were obtained from muscles of 2–3-month-old C57/BL6 male mice carrying the LacZ transgene in Rosa26 locus and cultured for 5 days. The SC-derived myoblast was cultured for 2 days, and then, the CD146+ cells were trypsinized and added to SC-derived myoblast culture in 1:1 ratio. The cells were co-cultured for 7 days in medium supporting myogenic differentiation, i.e., DMEM 1 g/l supplemented with 10% HS, 20% FBS, 1% penicillin/streptomycin, and 0.5% chicken embryo extract (CEE). Then, cells were fixed and analyzed.

SC-derived myoblasts cultured of 3 days, MIPCs cultured for 5 days, and BMSCs cultured for 7–10 days were tested for their ability to undergo adipogenic and chondrogenic differentiation. To this point, the culture medium was changed to AdipoMAX Differentiation Medium (Sigma-Aldrich) or ChondroMAX Differentiation Medium (Sigma-Aldrich), and cells were cultured for 2 or 7 days correspondingly in such differentiation medium.

Then, cells that were cultured in differentiating conditions were fixed with 3% paraformaldehyde (PFA) in PBS. The cells in myogenic differentiation medium were stained with Giemsa, and fusion index was assayed, or the skeletal muscle/myotube-specific myosin expression was verified using antibodies against skeletal myosin (description of immunocytochemistry assay below). In MIPC co-cultures with myoblasts, the expression of the skeletal muscle/myotube-specific myosin and β-galactosidase was verified. Cells in the adipogenic or chondrogenic medium were correspondingly stained with Oil Red O or Alcian Blue, according to manufacturer’s protocol. Three independent experiments were performed for each analyzed cell population.

### Heterotopic transplantation

5 × 10^5^ of CD146+ MIPCs, SC-derived myoblasts, or BMSCs were suspended in 0.5 ml of Matrigel GFR High Concentration (HC) Basement Membrane Matrix (Corning). Next, aliquots of approximately 0.4 ml of Matrigel with cells were injected in the subcutaneous tissue of the back of C57/BL6 male mice. After 21 days, transplants were isolated for further analysis. Three independent experiments were performed for each of the examined cell populations.

### Quantified real-time PCR

Total RNA was extracted from SC-derived myoblasts (3 days of culture), CD146+ MIPCs (5 days of culture), or BMSCs (7–10 days of culture) using High Pure Isolation Kit (Roche), according to manufacturer’s instruction. Then, cDNA based on isolated mRNA was synthesized using RevertAid First-Strand cDNA Synthesis Kit (ThermoFisher Scientific), in accordance to manufacturer’s protocol, under the following conditions: 25 °C for 5 min, 42 °C for 90 min, and 70 °C for 5 min. mRNA levels were assessed using quantitative real-time PCR analysis (qPCR) with TaqMan assays for the following genes: *Mcam (Cd146), Pax7, Myf5, Myod, myogenin, nestin, Runx2, Fap, Cxcr4, Pw1, Cspg4 (Ng2)*, *Tcf4, Pdgfrβ, Tbx18,* and *Alp*. The average expression of hypoxanthine phosphoribosyltransferase 1 (*Hprt1*) and glyceraldehyde-3-phosphate dehydrogenase (*Gapdh*) was used as reference gene expression for further calculations. The reaction was performed with TaqMan Gene Expression Master Mix (ThermoFisher Scientific) using LightCycler 96 (Roche) in following conditions: preincubation 2 min, 50 °C; preincubation 10 min, 95 °C; amplification (40 cycles) 15 s, 95 °C, and 1 min, 60 °C. All reactions were performed in duplicates. Expression levels were calculated with 2-(ΔΔCt) formula in reference to the relative expression of examined genes in 13.5-day-old mouse embryo. Three independent experiments were performed for each of the examined cell populations.

### Immunocytochemistry and immunohistochemistry

Skeletal muscle frozen sections (5 μm), transplants, and cells isolated from the muscle and attached to poly l-lysine (Merck) or in vitro cultured SC-derived myoblasts (3 days of culture), MIPCs (5 days of culture), CD146+ MIPCs (0 days of culture), CD146+ MIPCs (5 days of culture), BMSCs (7–10 days of culture), CD146+ BMSCs (7 days of culture), or fibroblasts (14 days of culture) were fixed with 3% PFA in PBS for 10 min. Next, specimens were washed in PBS and were permeabilized in 0.05% Triton X100 (Sigma-Aldrich) in PBS for 3 min. Further, specimens were washed in PBS and incubated in 0.25% glycine (Sigma-Aldrich) in PBS, followed by incubation in 3% bovine serum albumin (Sigma-Aldrich) with 2% donkey serum albumin (Sigma-Aldrich) in PBS for 1 h. Next, samples were incubated with primary antibodies (anti-Pax7, Developmental Studies Hybridoma Bank DSHB; anti-CD146, 134702, BioLegend; anti-Fap, ab53066, Abcam, anti-nestin, ab6142, Abcam; anti-nestin, ab81462, Abcam; anti-Runx2, ab76956, Abcam; anti-beta galactosidase, ab9361, Abcam; anti-CD34, ab8536, Abcam; anti-skeletal myosin, M7523, Sigma-Aldrich; anti-laminin, L9393, Sigma-Aldrich) diluted 1:50 (anti-Pax7) or 1:100 (other ones) in 3% BSA with 2% donkey serum in PBS at 4 °C overnight, followed by incubation in appropriate secondary antibodies conjugated with either Alexa Fluor 488 or 594 (anti-mouse, 21203; anti-rat, 21208; anti-rat, 11077; anti-rabbit 21206; anti-rabbit 21207; anti-goat, 21468; anti-chicken, 11039; ThermoFisher Scientific) diluted 1:500 in 1.5% BSA in PBS in room temperature for 2 h. Negative controls of secondary antibodies were performed. Cell nuclei were visualized by 5-min long incubation in Hoechst 33342 (ThermoFisher Scientific) diluted 1:1000 in PBS. Specimens were mounted with Fluorescent Mounting Medium (Dako Cytomation) and analyzed using confocal microscope LSM 700 (Zeiss) and ZEN software (Zeiss). The proportion of cells expressing examined proteins was calculated from 10 fields of view on each slide and each experiment was performed three times.

### Statistical analysis

The mean value and standard deviation were shown. The results were analyzed with a one-way ANOVA test and post hoc with Tukey’s multiple comparisons test.

## Results

### The localization and characterization of CD146+ muscle interstitial progenitor cells, satellite cell-derived myoblasts, and bone marrow stromal/stem cells

**Fig. 1:**
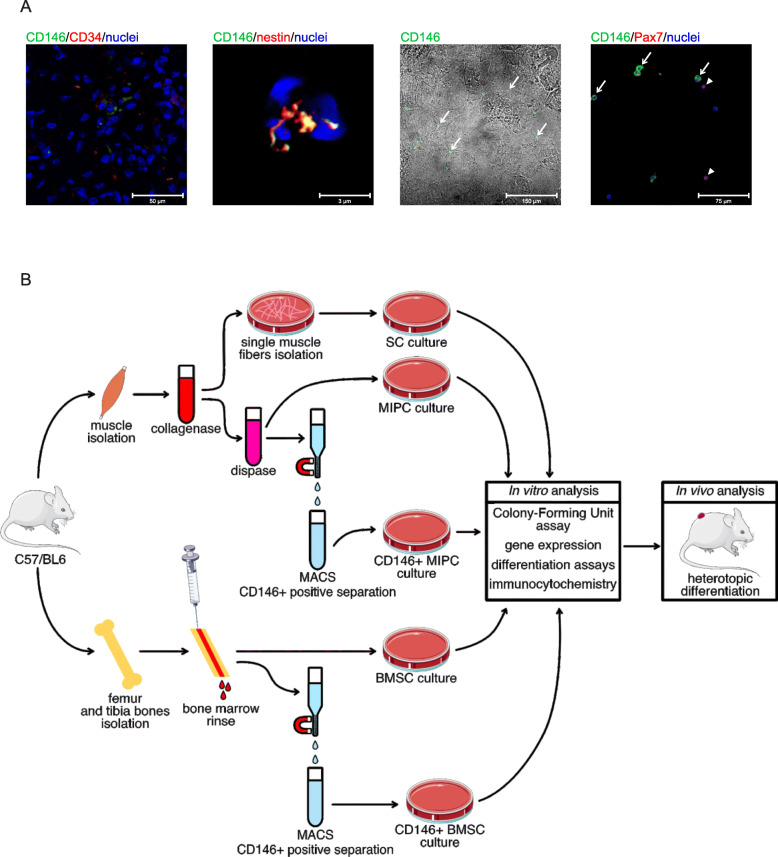
The isolation and localization of CD146+ MIPCs. **a** The localization of CD146 (green) and co-localization of CD146 (green) with CD34 (red) or nestin (red) in the mouse *Gastrocnemius* muscle, co-localization of CD146 (green), and PAX7 (red) in freshly isolated cells attached to poly-l-lysine covered slides; CD146+ cells marked with arrows, PAX7+ cells marked with arrowheads. **b** Experimental design

First, we localized CD146+ muscle interstitial progenitor cells (MIPCs) in the *Gastrocnemius* muscle (Fig. 1a). CD146+ cells were located near the CD34+ cells between the myofibers (Fig. 1a). CD34+ is expressed by different cell types including endothelial cells and endothelial progenitor cells. We observed the co-localization of CD146 and nestin in CD146+ MIPCs. Then, we analyzed cells that were freshly isolated from the muscle and attached to poly-l-lysine-covered slides. We observed the presence of two cell populations—one expressing CD146 in the cell membrane and second expressing PAX7 in the nucleus. We did not detect cells expressing both CD146 and PAX7 (Fig. 1a). To compare the number of MIPCs and SCs in the skeletal muscle, we counted the CD146+ cells and PAX7+ cells in muscle sections in which each antigen was immunolocalized. We noticed 4 +/− 3 PAX7+ cells and 6 +/− 3 CD146+ cells. Thus, the relation of SCs to MIPCs was 0.7:1.

**Fig. 2:**
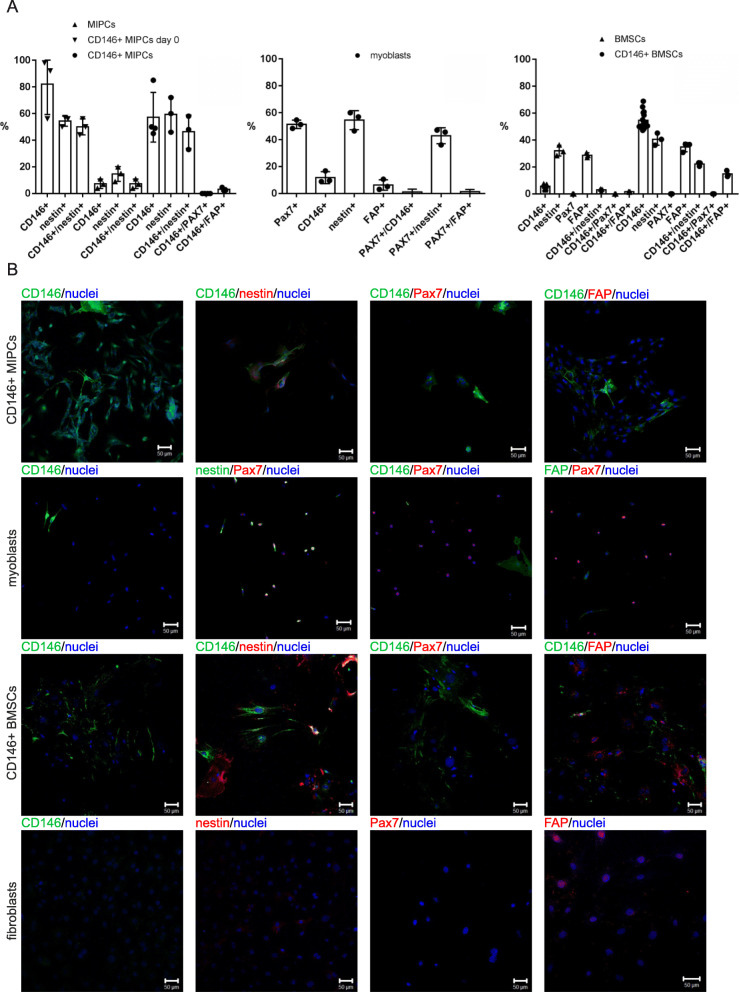
The characterization of MIPCs, SC-derived myoblasts and BMSCs. **a** The proportion of CD146, nestin, CXCR4, PAX7, and FAP-positive cells in non-sorted MIPCs cultured for 5 days, CD146+ MIPCs isolated and sorted directly from the muscle (day 0) or cultured for 5 days (CD146+ MIPCs), (*n* = 3–4); the proportion of CD146, nestin, CXCR4, PAX7, and FAP-positive cells in SC-derived myoblasts cultured for 3 days (*n* = 3); the percentage of CD146, nestin, CXCR4, PAX7, and FAP-positive cells in CD146+ BMSCs and whole population of BMSCs cultured for 7–10 days, (*n* = 3–15); **b** localization of selected markers: CD146; nestin, CXCR4, PAX7, and FAP in CD146+ MIPCs (day 5 of culture), SC-derived myoblasts (day 3 of culture), CD146+ BMSCs (day 7 of culture), and fibroblasts (day 14 of culture), blue - nuclei; green - CD146, nestin; red - nestin, CXCR4, PAX7, FAP, scale bar 50 μm, (*n* = 3)

Next, we isolated different populations of cells (Fig. 1b) including (1) freshly isolated and sorted CD146+ MIPCs (day 0), (2) whole population of MIPCs (unsorted cells), (3) CD146+ MIPCs sorted out of the cells isolated from hind limb skeletal muscles and cultured for 5 days, (4) myoblasts derived from SCs isolated from single myofibers from hindlimb skeletal muscles and cultured for 3 days, (5) whole population of BMSCs isolated from the bone marrow and cultured for 7 days, and (6) CD146+ BMSCs isolated from the bone marrow, sorted and cultured for 7 days. First, we analyzed the presence of proteins characteristic for different populations of stem and progenitor cells, such as CD146, nestin, PAX7, and fibroblast activation protein-α (FAP) (Fig. 2). The CD146 was described as a marker of human muscle clonogenic myogenic progenitors different from satellite cells and also of human bone marrow stem cells, to distinguish them from stromal cells [28, 30]. Nestin was defined as a marker of mouse bone marrow stem cells and muscle satellite cells [31–34]. PAX7 is a well-known marker of satellite cells [5]. The whole population of MIPCs contained 7.3% +/− 3.5 of CD146+ cells and 100% of these cells expressed nestin (Fig. 2a). The sorted population of MIPCs was significantly enriched in CD146+ cells. Freshly isolated and sorted cells contained 82% +/− 22.7 of CD146+ MIPCs and 57.3% +/− 18.6 after 5 days of culture. Correspondingly, 63.5% +/− 14 and 81.3% +/− 13.3 of them expressed nestin. PAX7 was not detected in CD146+ MIPCs. We noticed that 3.2% +/− 1.3 of CD146+/FAP+ cells in CD146+ MIPC culture (Fig. 2a). Thus, the population of CD146+ MIPCs could be described as CD146+/nestin+/PAX7−/FAP−. Analysis of SC-derived myoblasts showed that 11.8% +/− 4.4 of them expressed CD146, but these cells did not express PAX7. Next, 51.4% +/− 3.1 of SC-derived myoblasts expressed PAX7 and 99.8% +/− 0.3 of them expressed nestin. The 5.1% +/− 7.2 of PAX7+ cells expressed FAP. Thus, SC-derived myoblasts were PAX7+/nestin+/CD146−/FAP−. The whole population of BMSCs contained 5.6 +/− 1.6 of CD146+ cells, 32.2% +/− 4 of nestin+ cells, 22.5% +/− 4.7 of RUNX2+ (Runt-related transcription factor 2, characteristic for osteogenic progenitors) cells, and 28.8% +/− 1.8 of FAP+ cells (Fig. 2, S1). The population of CD146+ BMSCs contained 54.7 +/− 6.5 of CD146+ cells, 40.6% +/− 4.5 of nestin+ cells, 11.1% +/− 3.4 of RUNX2+ cells, and 34.8% +/− 3.4 of FAP+ cells (Fig. 2, S1). Moreover, PAX7 expressing cells were noticed neither in BMSCs nor in CD146+ BMSC population (Fig. 2, S1). The 45.3% +/− 3.8 of CD146+ BMSCs expressed nestin and 3.1% +/− 2.3 expressed RUNX2 (Fig. 2, S1). CD146+ BMSCs did not express PAX7; however, 27.1% +/− 4.1 of them expressed FAP. Thus, CD146+ BMSCs were CD146+/nestin+/−/PAX7−/RUNX2−.

**Fig. 3:**
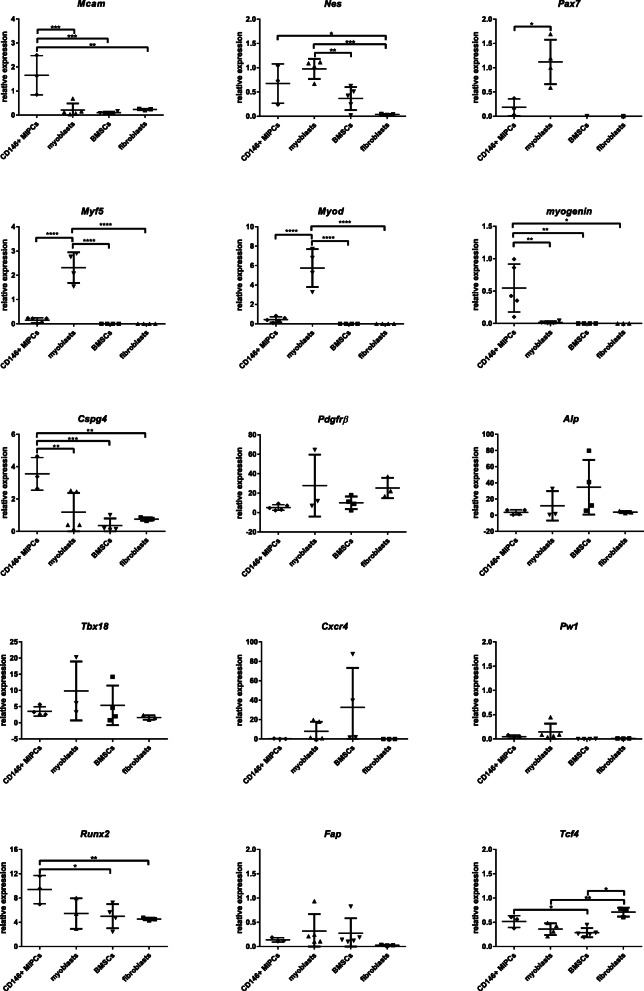
The characterization of MIPCs, SC-derived myoblasts, and BMSCs. The mean value and standard deviation were shown. The results were analyzed with a one-way ANOVA test and post hoc with Tukey’s multiple comparisons test (*p < 0.05; **p < 0.005; ***p < 0.0005). The level of selected marker mRNA expression in CD146+ MIPCs (day 5 of culture), SC-derived myoblasts (day 3 of culture), whole population of BMSCs (day 7 of culture), and fibroblasts (day 14 of culture) (*n* = 3–5)

Then, we followed the level of mRNAs, encoding such markers as *melanoma cell adhesion molecule* (*Mcam* coding CD146), *Nes* (coding nestin), *Pax7, Myf5, Myod, myogenin, Cspg4* (coding NG2)*, Pdgfrβ, Alp, T-box transcription factor 18 (Tbx18), Cxcr4, Pw1, Runx2, Fap,* and *transcription factor 4* (*Tcf4*) in CD146+ MIPCs, SC-derived myoblasts, whole population of BMSCs, and mouse primary fibroblasts (Fig. 3, S1). The expression of *Mcam* was the highest in CD146+ MIPCs. The differences between the levels observed in CD146+ MIPCS, SC-derived myoblasts, BMSCs, and fibroblasts were significant. Expression of *Nes* was comparable in CD146+ MIPCs and SC-derived myoblasts and lower in BMSCs, and it was very low in fibroblasts. The *Pax7* mRNA was detected only in SC-derived myoblasts. However, the low level of *Pax7* mRNA was present also in CD146+ MIPCs. Similarly, expression of myogenic regulatory factor (MRF) characteristic for early stages of myoblast differentiation, i.e., *Myf5* and *Myod*, was detected only in SC-derived myoblasts. Interestingly, we detected low level of *myogenin* expression in CD146+ MIPCs. Next, we analyzed expression of pericyte markers, i.e., *Cspg4*, *Pdgfrβ*, *Alp*, and *Tbx18* (Fig. 3). The level of *Cspg4* was significantly higher in CD146+ MIPCs, as compared to SC-derived myoblasts, BMSCs, and fibroblasts. However, the level of other markers, such as *Pdgfrβ*, *Alp*, and *Tbx18* was similar in all analyzed cells. We did not detect the significant differences in *Cxcr4* expression between examined cell populations. Then, we followed the level of PICs marker, i.e., *Pw1* and *Runx2* (Fig. 3, S1). *Pw1* expression was very low in CD146+, SC-derived myoblasts, BMSCs, and fibroblasts. Interestingly, *Runx2* mRNA expression was higher in CD146+ MIPCs, then in SC-derived myoblasts, BMSCs, or fibroblasts. Next, we compared the level of mRNA encoding fibroblast marker—FAP (Fig. 3). The expression of *Fap* was comparable in CD146+ MIPCs, SC-derived myoblasts, BMSCs, and primary fibroblasts. Finally, we analyzed the expression of another fibroblasts marker, i.e., *Tcf4*, which is strongly expressed in connective tissue fibroblasts [35]. Indeed, the highest expression of this mRNA was observed in fibroblasts.

### In vitro differentiation of CD146+ muscle interstitial progenitor cells, satellite cell-derived myoblasts, and bone marrow stromal/stem cells

**Fig. 4:**
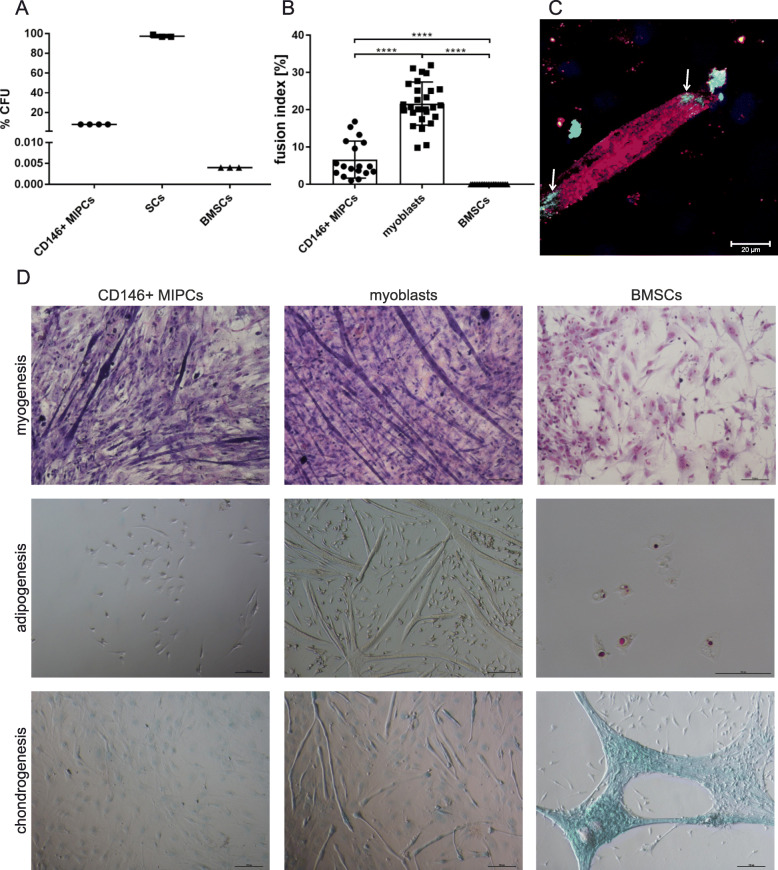
The in vitro differentiation of CD146+ MIPCs, SC-derived myoblasts, and BMSCs. **a** The percent of CFU (*n* = 3–4); **b** fusion index; **c** hybrid myotubes in CD146+ MIPC co-cultures with myoblasts, MIPCs (β-galactosidase green) incorporated to myotubes (myosin - red, nuclei - blue) marked with arrows; **d** the culture in myogenesis, adipogenesis, and chondrogenesis promoting medium, myotubes (Giemsa staining), adipoblasts (Oil redO staining), and chondroblasts (Alcian Blue staining)

First, we performed CFU assay to evaluate the presence of cells capable to form clones, i.e., cells fulfilling the criteria of progenitor cells. We noticed that clones were formed by 8% of CD146 + MIPCs, 97.6% +/− 1.2 of SC-derived myoblasts, and 0.004% of BMSCs (Fig. 4a). Thus, the clonogenic potential of SC-derived myoblasts was the highest. Next, we analyzed the ability of CD146+ MIPCs, SC-derived myoblasts, and BMSCs to differentiate in vitro, in media inducing either myoblasts, or adipoblasts, or chondroblast differentiation (Fig. 4b–d). First, we analyzed the spontaneous/naïve potential of CD146+ MIPSCs, SC-derived myoblasts, and BMSCs to fuse and form myotubes in myogenic medium (Fig. 4b, d). The fusion index, depicting how many analyzed cells participated in the formation of myotubes, differed significantly depending on cell type. In case of SC-derived myoblasts, fusion index was 21.66% +/− 5.7 and 6.66% for +/− 5.0 CD146+ MIPCs (Fig. 4b). CD146+ MIPCs and SC-derived myoblasts fused spontaneously (Fig. 4d). The morphology of myotubes did not differ between SC-derived myoblasts and MIPCs. We did not detect myotubes formed from BMSCs (Fig. 4b, d). Moreover, we showed that CD146+ MIPCS could fuse with myoblasts in co-cultures; thus, we were able to detect hybrid myotubes in such cultures (Fig. 4c). The CD146+ MIPCs were identified on the basis of β-galactosidase expression, and the myotubes were localized on the basis of skeletal myosin presence.

Culture in adipogenic differentiation medium resulted in the presence of adipocytes stained with Oil RedO only in BMSC cultures. Under such culture conditions, CD146+ MIPCs did not follow adipogenic differentiation; thus, we were not able to detect cells stained with Oil RedO. Interestingly, SC-derived myoblasts formed myotubes also in adipogenic differentiation medium. Chondrogenic differentiation was observed in the case of BMSCs, but not in the case of CD146+ MIPCs or SC-derived myoblast cultures (Fig. 4c). SC-derived myoblasts cultured in chondrogenic differentiation medium formed myotubes as well. CD146+ MIPCs did not fuse with each other and also did not undergo chondrogenic differentiation when cultured in an appropriate medium. Thus, we showed that CD146+ MIPCs and SC-derived myoblasts followed myogenic differentiation and BMSCs presented chondrogenic and adipogenic, but not myogenic potential in vitro.

### In vivo differentiation of CD146+ muscle interstitial progenitor cells

**Fig. 5:**
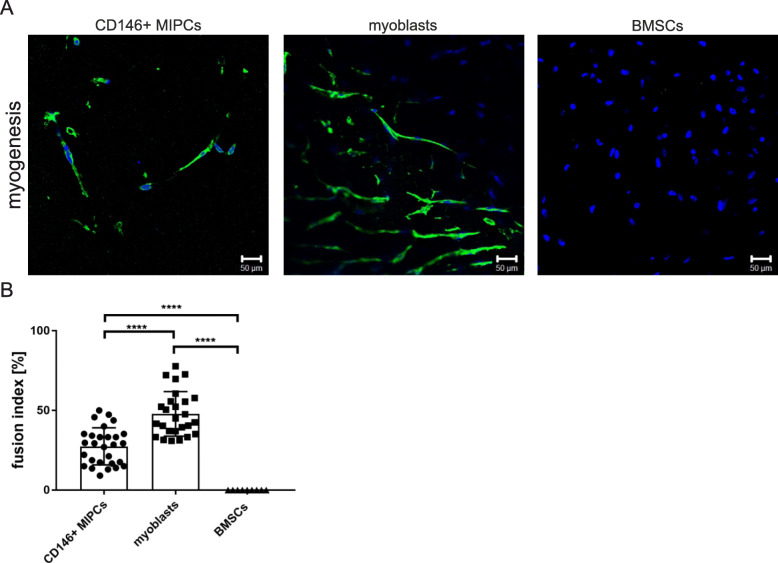
The in vivo myogenic differentiation of CD146+ MIPCs, SC-derived myoblasts, and BMSCs. Cells were cultured in vitro and then transplanted subcutaneously in Matrigel. After 21 days the transplants were isolated and analyzed. **a** Localization of myosin (green), nuclei (blue); **b** fusion index

To follow the ability of CD146 + MIPSCs, SC-derived myoblasts and BMSCs to undergo in vivo differentiation, we subcutaneously transplanted Matrigel containing these cells (Fig. 5). We showed that under such conditions, CD146+ MIPCs were able to fuse in the absence of exogenous myoblasts. We detected the presence of myotubes expressing myosin. The myotubes were also detected in myoblasts transplants but not in BMSCs. The fusion index of MIPCs was 27.5% +/− 11.6, and of SC-derived myoblasts was 47.9 +/− 14.0 (Fig. 5). Thus, similarly to in vitro culture, MIPCs showed the ability to form myotubes, however, with lower efficiency than SC-derived myoblasts. However, we proved that CD146+ MIPCs and myoblasts presented naïve myogenic potential in vivo.

## Discussion

The skeletal muscle interstitium accompanies myofibers and is important to retain skeletal muscle homeostasis [36]. Many types of cells such as FAPs, resident myeloid cells, fibroblasts, and vascular endothelial cells, exist in skeletal muscle interstitium [12, 37, 38]. Moreover, different populations of progenitor cells could be found there [12, 37, 38]. In the current study, we used the CD146 marker to isolate the population of mouse muscle interstitial progenitor cells (MIPCs). This marker was used by Sacchetti and co-workers to isolate stem cells from human bone marrow and progenitor cells from human muscles [28, 30, 39] and by Crisan and co-workers to isolate perivascular mesenchymal cells from different human tissues [40]. It was shown that human CD146+ cells isolated from the bone marrow represented the subendothelial, perivascular stem cell population, able to self-renew and differentiate into the bone and form hematopoietic microenvironment, i.e., bone marrow, after their subcutaneous transplantation into immunocompromised mice [28, 39]. The CD146+ cells from human muscles were found to be associated with microvessels and presented clonogenic and myogenic, but not osteogenic potential [28, 30, 40]. However, it was also shown that human muscle CD146+ cells were also sclerogenous [40]. The described population of CD146+ cells was named “mesenchymal stem cells” and was compared to pericytes [28, 30, 39].

In our study, mouse CD146+ MIPCs were isolated from the suspension of muscle-derived cells. MIPCs did not express PAX7, i.e., satellite cell marker and FAP, i.e., fibroblast marker. Majority of the freshly isolated CD146+ MIPCs expressed nestin. During in vitro culture, some of the cells lost CD146 but almost all of them expressed nestin. The decrease of CD146 expression was also observed by others in in vitro cultured human muscle CD146+ cells [28]. Nestin, which is an intermediate filament protein and which expression was shown in few populations of mouse muscle cells, such as satellite cells [31] and pericytes [24], decreases following satellite cell activation and differentiation [31]. The lack of functional nestin accelerated myoblast differentiation and overexpression of nestin and their inhibited differentiation [41]. It was documented that nestin regulates Cdk5/p35 signaling complex [41, 42]; however, the role of nestin in MIPC proliferation and differentiation was not studied, yet. Our analysis showed that CD146+ MIPCs expressed nestin at a similar level as SC-derived myoblasts. Importantly, mouse CD146+ MIPCs expressed *Cspg4* mRNA (coding NG2) at a higher level than SC-derived myoblasts, BMSCs, and fibroblasts. NG2 is routinely used as the marker of pericytes localized in mouse skeletal muscles, and it is not detected in quiescent SCs or FAPs [18, 21, 24, 25, 43, 44]. Thus, we suggested that MIPCs could be a population of pericytes present in skeletal muscles. However, mRNAs of other known muscle pericyte markers, such as *Pdgfrβ, Tbx18,* and *Alp*, used previously to detect mouse pericytes present in the skeletal muscles [19, 21, 43], were expressed at the same level in CD146+ MIPCs, SC-derived myoblasts, BMSCs, and fibroblasts. CD146 was also detected in the pericytes isolated from mouse skeletal muscles on the basis of TBX18 or NG2 presence [21, 24, 43]. Next, mouse CD146+ MIPCs analyzed by us did not express *Cxcr4* and *Pw1* mRNA. CXCR4 is a stromal-derived factor - 1 (SDF-1) receptor, which is present in several types of stem and progenitor cells [45, 46]. PW1 is a transcription factor synthesized by SCs and PICs [47]. The level of fibroblast marker, such as *Tcf4*, was lower than in adult fibroblasts and FAP level was lower than in SC-derived myoblasts and BMSCs. Thus, we concluded that CD146+ MIPCs could be a pericyte and that they differ from SCs, FAPs, and fibroblasts.

Further analysis showed that a portion of CD146+ MIPCs presented clonogenic and myogenic potential. Myogenic differentiation of CD146+ MIPCs was showed by us in a few experiments. First, these cells co-cultured with exogenous myoblast were able to fuse them. Second, when cultured under conditions promoting myogenic differentiation CD146+ MIPCs were able to spontaneously fuse with each other. Moreover, we detect the expression of *myogenin* in CD146+ MIPCs cultured for 5 days. However, their process was less effective, as compared to SC-derived myoblasts. Heterotopic transplantation of CD146+ MIPCs allowed us to show that they are also able to follow the myogenic program in vivo. Thus, they showed similar properties to CD146+ cells isolated from human skeletal muscles [28, 30, 40]. Crisan and co-workers reported the presence of myogenic CD146+ cells, described as “mesenchymal stem cells,” associated with microvessels of the skeletal muscles and other tissues [40]. These cells expressed also NG2, PDGFRβ, and “mesenchymal stem cell” markers, such as CD73, CD90, and CD105, and were able to follow an adipogenic, osteogenic, and chondrogenic program in vitro [40]. Cultured under myogenesis promoting conditions, they were able to fuse with each other and after intramuscular transplantation into regenerating muscle to participate in new myofiber formation [40]. The human muscle CD146+ cells, described by Sacchetti and co-workers, also expressed “mesenchymal stem cell” markers, such as CD73, CD90, and CD105. These cells were perivascularly located, similarly to pericytes, were characterized by the clonogenic and myogenic potential in vitro and in vivo, i.e., after their intramuscular and ectopic transplantation [28, 30]. Interestingly, human muscle CD146+ cells guided and organized the formation of blood vessels in co-cultures with endothelial cells [28]. However, under such conditions, the myogenesis of CD146+ cells was inhibited, suggesting that blood vessel formation and myogenesis are alternative fates. Importantly, the chondrogenic or osteogenic differentiation of human muscle CD146+ cells was not observed in vivo after subcutaneous transplantation of these cells [30]. Correspondingly, we did not observe adipogenic or chondrogenic differentiation of mouse CD146+ MIPCs. However, we detected an increased level of *Runx2* mRNA, i.e., a transcription factor crucial for osteogenesis [48], comparing to SC-derived myoblasts, BMSCs, and fibroblasts in that the level of this transcript was low. We also noticed important differences between naïve differentiation potential between muscle and bone marrow cells that did not spontaneously follow the myogenic program but were able to undergo adipogenesis and chondrogenesis. Interestingly, SC-derived myoblasts formed myotubes independently of the type of culture medium used, while myogenic differentiation of mouse CD146+ MIPCs was observed only in medium-stimulating myogenic differentiation but not chondrogenic or adipogenic ones. It suggests that CD146+ MIPC myogenic differentiation could be induced by exogenous signals present in a myogenic culture medium.

The properties of human and mouse muscle CD146+ cells could be compared with human and mouse muscle pericytes. Human pericytes associated with microvasculature fused when in vitro cultured in myogenesis inducing medium and also were able to differentiate into osteoblasts or adipoblasts [18]. After transplantation, these cells participated in the formation of new myofibers in an immunodefficient dystrophic mice [18]. Mouse endogenous (not transplanted) ALP+ pericytes fused with differentiating myofibers and were able to settle SC niche during post-natal growth and to participate in skeletal muscle regeneration [19]. However, it was also shown that endogenous pericytes, localized on the basis of TBX18 presence, did not participate in new myofiber formation in vivo [43]. Nevertheless, human and mouse pericytes promoted post-natal growth and satellite cell quiescence through insulin-like growth factor and angiopoietin-1 (ANGPT-1) [21]. Recently, modulation of Notch and PDGF pathways induced perivascular cell features of mouse and human SCs [49]. Activation of these pathways increased the migration ability of SCs in vitro and in vivo what could eventually improve the therapeutic potential of these cells [49]. Thus, the interaction between SCs and pericytes seems to be complex and plays a very important role in muscle homeostasis.

## Conclusions

In the current study, we showed that mouse muscle-derived CD146+ cells represent the population of mouse muscle interstitial progenitor cells that could be a population of pericytes and that differ from satellite cell-derived myoblasts and CD146 + BMSCs. Mouse muscle-derived CD146+ cells could follow the myogenic program in vitro and in vivo. Mouse CD146+ MIPCs present similar properties to previously described human CD146+ clonogenic and myogenic progenitors [28, 30, 40]. We suggest that these cells could be considered as a source of cells for muscle cell therapy.

## Supplementary information

**Additional file 1: Figure S1.** A - the proportion of RUNX2 and CD146 positive cells; B - localization of CD146+ (green) and RUNX2 (red) in BMSCs

## Data Availability

Department of Cytology, Faculty of Biology, University of Warsaw, Miecznikowa 1 St, 02-096 Warsaw, Poland.

## Abbreviations

ALP: Alkaline phosphatase
ANGPT-1: Angiopoietin-1
BMSC: Bone marrow-derived mesenchymal stromal/stem cell
CEE: Chicken embryo extract
CFU: Colony-forming unit
CXCR4: C-X-C chemokine receptor type 4 (SDF-1 receptor)
DMEM: Dulbecco’s modified Eagle’s medium
FAP: Fibro-adipogenic progenitor
FAP: Fibroblast activation protein-α
FBS: Fetal bovine serum
GFR: Growth factor reduced
HPRT1: Hypoxanthine phosphoribosyltransferase 1
HS: Horse serum
IGF: Insulin-like growth factor
MCAM: Melanoma cell adhesion molecule (CD146)
MIPC: Muscle interstitial progenitor cell
MRF: Myogenic regulatory factor
NG2: Neural-glial antigen 2
NCAM: Neural cell adhesion molecule (CD56)
PBS: Phosphate-buffered saline
PIC: PW1+ interstitial cell
PDGFR: Platelet-derived growth factor receptor
RUNX2: Runt-related transcription factor 2
Sca1: Stem cell antigen-1
SC: Satellite cell
SDF-1: Stromal-derived factor-1
αSMA: Smooth muscle α-actin
TCF4: Transcription factor 4
TBX18: T-box transcription factor 18
VCAM: Vascular cell adhesion protein (CD106)
