# Supplementary figures and images for: Mouse CD146+ muscle interstitial progenitor cells differ from satellite cells and present myogenic potential
Source: Stem Cell Res Ther. 2020 Aug 6;11:341. doi: 10.1186/s13287-020-01827-z (PMC7409690; doi:10.1186/s13287-020-01827-z)

A

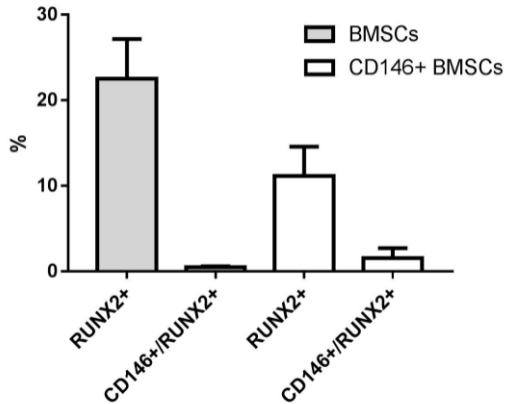

B

CD146/RUNX2/nuclei

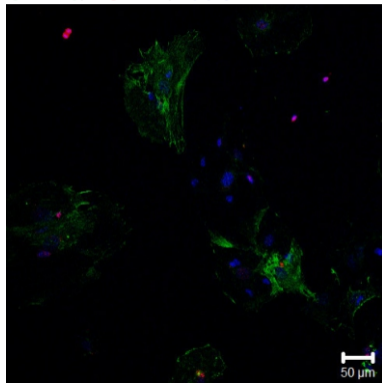

Supplement: Supplementary file 1 — Additional file 1: Figure S1. A - the proportion of RUNX2 and CD146 positive cells; B - localization of CD146+ (green) and RUNX2 (red) in BMSCs [file 13287_2020_1827_MOESM1_ESM.pdf]
